# *In-vivo* turnover frequency of the cyanobacterial NiFe-hydrogenase during photohydrogen production outperforms *in-vitro* systems

**DOI:** 10.1038/s41598-018-24430-y

**Published:** 2018-04-17

**Authors:** Kirstin Gutekunst, Dörte Hoffmann, Ulrike Westernströer, Rüdiger Schulz, Dieter Garbe-Schönberg, Jens Appel

**Affiliations:** 1 Botanical Institute, Christian-Albrechts-University, 24118 Kiel, Germany; 2 Institute of Geosciences, Christian-Albrechts-University, 24118 Kiel, Germany

## Abstract

Cyanobacteria provide all components for sunlight driven biohydrogen production. Their bidirectional NiFe-hydrogenase is resistant against low levels of oxygen with a preference for hydrogen evolution. However, until now it was unclear if its catalytic efficiency can keep pace with the photosynthetic electron transfer rate. We identified NikKLMQO (sll0381-sll0385) as a nickel transporter, which is required for hydrogen production. ICP-MS measurements were used to quantify hydrogenase molecules per cell. We found 400 to 2000 hydrogenase molecules per cell depending on the conditions. *In-vivo* turnover frequencies of the enzyme ranged from 62 H_2_/s in the wild type to 120 H_2_/s in a mutant during photohydrogen production. These frequencies are above maximum *in-vivo* photosynthetic electron transfer rates of 47 e^−^/s (equivalent to 24 H_2_/s). They are also above those of existing *in-vitro* systems working with unlimited electron supply and show that *in-vivo* photohydrogen production is limited by electron delivery to the enzyme.

## Introduction

Biohydrogen production offers an appealing alternative to fossil fuel burning. It does not involve the production of toxic side products and is CO_2_-neutral. The different hydrogenases (Fe-, FeFe-, and NiFe-) taking part in biological hydrogen turnover evolved into extremely efficient catalysts since the primordial earth^1–3^. Many different options for biohydrogen evolution are available and are studied such as fermentative hydrogen production^4^, direct biophotolysis of water^5,6^, and exploitation of microbial consortia^7^. Direct biophotolysis is the most energy efficient process since it is converting sunlight and water directly to O_2_ and H_2_ without organic intermediates. However, it faces the challenge that the FeFe-hydrogenases, which are considered the most efficient H_2_-producing enzymes, are working specifically under anaerobic conditions and are inactivated by oxygen.

Cyanobacteria perform oxygenic photosynthesis and harbor two types of NiFe-hydrogenases^8^. Protein film voltammetry experiments showed that the cyanobacterial bidirectional NiFe-hydrogenase has a bias to hydrogen production^9^, which was considered uncommon for NiFe-enzymes. In addition, this hydrogenase is reduced by the low potential electron donors ferredoxin and flavodoxin^10^ supporting its role as H_2_-producer. Since this enzyme remains partially active in the presence of low concentrations of oxygen^9^ it is a promising candidate for biotechnological photohydrogen production. One of the major questions to be solved in this respect is if the hydrogen turnover of the cyanobacterial NiFe-hydrogenase is able to cope with the rate of photosynthetic electron transport. However, the rate of hydrogen formation at its active site is not known. We therefore developed an approach to first quantify the number of hydrogenase molecules in the cells. On this basis the turnover rates for hydrogen production of the enzyme could be determined.

The assembly of metalloenzymes such as hydrogenases requires a complex array of additional proteins^11^. Accessory proteins ensure that the correct metal is inserted into the active site. This is owing to the so-called Irving-Williams series of the affinity of the transition metals to whatever ligand. Predominantly due to their decreasing ionic radii and the increasing electrostatic effect the affinity increases in the order Mg^2+^  < Mn^2+^  < Fe^2+^  <Co^2+^  <Ni^2+^  <Cu^2+^ > Zn^2+ ^^12^. Cells are thus facing a problem. They need to avoid that a wrong metal is inserted, which might be bound with a higher affinity but does not confer catalytic activity. They tackle this challenge by expressing a number of metal-binding accessory proteins that channel the correct metal into the proper active site not allowing these metals to escape from these routes^13,14^. As soon as the respective metal-ion is part of the folded holoenzyme the chance to be exchanged becomes negligible at least inside the cell. In addition, metal ion homeostasis inside the cells is tightly controlled according to their binding affinities so that they are not able to supersede metals in inappropriate active sites. In brief, cells should contain the number of metal ions that correspond to the number of appropriate binding sites including enzymes and storage proteins.

Ni-enzymes are an intriguing example of these mechanisms and recent years saw a considerable increase in our understanding of how they acquire nickel^11,15^. Especially, active NiFe-hydrogenases are the product of the sophisticated interaction of a minimum of 8 accessory proteins and a nickel transporter^16,17^.

In this study we used the model cyanobacterium *Synechocystis* sp. PCC 6803. It harbors a single NiFe-hydrogenase that produces hydrogen under fermentative conditions as well as photohydrogen when cells are shifted from anaerobic conditions into the light^10,18–20^. This NiFe-hydrogenase and the urease are the only known nickel enzymes in *Synechocystis*^21^.

Since the nickel transporter necessary for the expression of an active hydrogenase remained elusive we deleted the putative transporter *nikKLMQO* (Δ*nik*) to unravel its specificity. ICP-MS was used to determine the number of nickel ions per cell in wild type cells, the hydrogenase deletion strain (Δ*hoxH*) and the hydrogenase overexpression strain (*oehox*). On this basis we were able to determine for the first time the number of hydrogenase molecules per cell and to deduce the *in-vivo* turnover frequency of the enzyme in different strains. This allowed us to compare the efficiency of cyanobacterial *in-vivo* photohydrogen production with optimized *in-vitro* systems of FeFe- and NiFe-hydrogenases from heterotrophic organisms that were tethered to PSI^22,23^. Knowledge about the enzymatic characteristics of the cyanobacterial NiFe-hydrogenase is basic to evaluate the feasibility of biotechnological approaches with this enzyme.

## Results

### Characterization of Δ*nik* mutant

**Figure 1:**
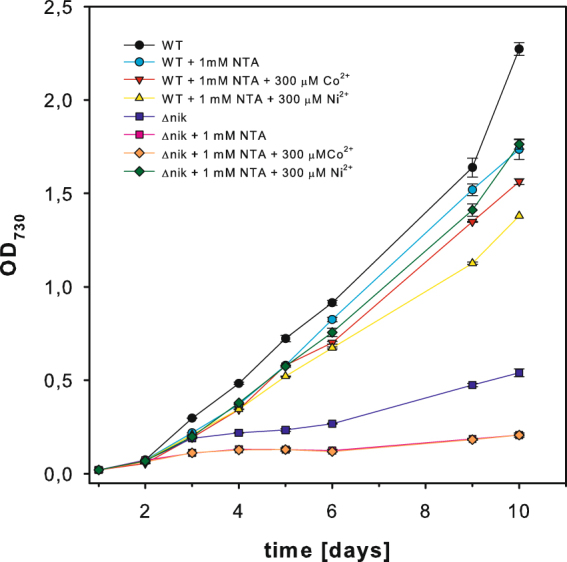
Growth curves of wild type cells and Δ*nik* in BG-11 and BG-11 with 1 mM NTA alone or 1 mM NTA and either 300 µM Co^2+^ or 300 µM Ni^2+^. Growth of Δ*nik* can be rescued upon addition of NTA and 300 µM Ni^2+^, which allows for unspecific uptake of nickel. In each case three independent cultures were measured in triplicates. The standard deviations are indicated and are smaller than the data points if not visible. Please note that the curves of Δ*nik* with 1 mM NTA and Δ*nik* with 1 mM and 300 µM Co^2+^ are on top of each other.

The putative nickel transporter NikKLMQO (*sll0381–sll0385*) was deleted from the genome of *Synechocystis* and complete segregation was checked by PCR and Southern blotting (s. Fig. S1). Wild type cells and Δ*nik* were grown in media with and without NTA and with supplements of Co^2+^ or Ni^2+^ (Fig. 1). NTA is a very efficient chelator of nickel and cobalt and therefore lowers the concentration of the free metal ions in the growth medium. Consequently, a high affinity transporter is necessary for their uptake. If NTA is added in combination with high concentrations (300 µM) of nickel or cobalt it masks these metals and it is thus possible to increase their concentration above otherwise toxic levels. This large supply enables the cells to unspecifically take up these metals via other metal transporters.

The Δ*nik* mutant grows significantly slower than wild type cells under normal conditions. If the availability of nickel is further decreased by addition of NTA (+1 mM NTA and +1 mM NTA + 300 µM Co^2+^) Δ*nik* cells loose their ability to grow. However, the growth phenotype of Δ*nik* can be rescued by addition of 1 mM NTA and 300 µM Ni^2+^, which facilitates unspecific nickel uptake. Addition of 300 µM Co^2+^ did not have any effect. This strongly suggests that *nikKLMQO* (sll0381, sll0382, sll0383, sll0384, and sll0385) encodes a nickel uptake system in *Synechocystis*.

### Hydrogenase and urease activity

**Figure 2:**
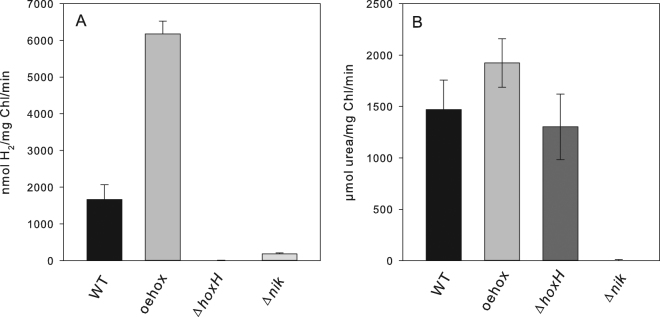
Hydrogenase (**A**) and urease (**B**) activities of the different strains used in this study. The activities of three independent cultures were measured in triplicates. The standard deviation is indicated.

*Synechocystis* possesses two known nickel-containing enzymes: a bidirectional NiFe-hydrogenase and a urease. In order to further characterize the Δ*nik* mutant and to investigate the nickel dependency of both enzymes, samples were taken from wild type cells, Δ*hoxH*, *oehox*, and Δ*nik* grown under normal conditions and subjected to measurements of hydrogenase and urease activity (Fig. 2) Hydrogenase activity was determined by adding the artificial electron donor methylviologen, which allows the quantification of functional hydrogenase in these cells. Δ*nik* shows only about 1/10^th^ of the hydrogenase activity of the wild type cells (Fig. 2A) and its urease activity is not detectable under the assay conditions used (Fig. 2B). These results further support the assumption that *nikKLMQO* codes for a nickel transporter.

### Metal content of different *Synechocystis* strains

In order to quantify the metal content of wild type cells, Δ*hoxH*, *oehox*, and Δ*nik* their cells were harvested, washed with EDTA, oxidatively broken down and subjected to ICP-MS. Table 1 shows the number of metal atoms per cell. The obtained values were in good agreement with published data as described in the following in detail.

**Table 1:** The number of metal atoms detected in the cells of the different strains used in this study.

Strain	Mg × 10^−7^	P × 10^−8^	S × 10^−8^	Ca × 10^−6^	Mn × 10^−5^	Fe × 10^−6^	Co × 10^−4^	Ni × 10^−4^	Cu × 10^−5^	Zn × 10^−5^	Mo × 10^−2^
Wild type	8.00 ± 0.49	3.50 ± 0.22	2.90 ± 0.14	5.20 ± 0.65	3.60 ± 0.55	6.20 ± 0.22	1.80 ± 0.22	1.50 ± 0.21	2.60 ± 0.16	4.10 ± 0.19	6.2 ± 1.2
ΔhoxH*	4.80 ± 0.51	3.00 ± 0.10	2.90 ± 0.08	2.80 ± 0.82	2.80 ± 0.46	7.40 ± 0.13	2.90 ± 0.17	1.30 ± 0.12	2.50 ± 0.14	2.90 ± 0.23	n.d.
oehox	7.70 ± 0.51	3.70 ± 0.30	3.30 ± 0.34	8.30 ± 4.10	4.00 ± 0.47	7.40 ± 0.48	1.90 ± 0.07	1.70 ± 0.14	2.40 ± 0.33	4.10 ± 0.33	n.d.
Δ*nik*	13.00 ± 3.23	5.40 ± 1.36	5.20 ± 1.32	22.00 ± 6.40	5.20 ± 1.34	12.00 ± 3.36	3.80 ± 1.05	0.40 ± 0.24	4.60 ± 1.28	9.10 ± 7.39	n.d.

*The values of the Δ*hoxH* were corrected for the larger cell size measured of the cells (supplementary data).

Of the metals measured magnesium is the most abundant. Since every chlorophyll molecule contains one magnesium atom we plotted the number of magnesium atoms per cell versus the number of chlorophyll molecules (s. Fig. S2). This yields a linear relationship with a y-axis intercept at about 4.36 × 10^7^ atoms per cell. This is the number of magnesium atoms not bound to chlorophyll and corresponds in the wild type with a cell size of 3.1 µm^3^ to a concentration of 21 mM. This is well in the range of magnesium concentration of 15 to 25 mM found in cells^24^.

Most of the phosphorus in the cells resides in RNA that makes up about 20% of their dry weight^25^. There are 3.5 × 10^8^ P-atoms in a wild type cell (Table 1). 20% of their dry weight would correspond to about 3.3 × 10^8^ P-atoms in RNA leaving sufficient P-atoms for the DNA and the different nucleotides (ATP, GTP, ADP, GDP etc.).

The sulfur content of a cell gives a direct measure of its protein content since 5% of all amino acids are methionine and cysteine in an average proteome^26^. A plot of the S-atoms per cell versus the P-atoms per cell shows a linear relationship (s. Fig. S3) suggesting a constant ratio of protein to RNA for the different strains as also described by the Redfield ratio for phytoplankton^27^.

Previous investigations found 4.5 × 10^5^ Mn-atoms per cell in Mn-limited cultures of *Synechocystis*^28^ and 4.2 × 10^6^ Fe-atoms in normal cultures^29^. In our case these values are in a similar range with 3.6 × 10^5^ Mn and 6.2 × 10^6^ Fe. Probably small variations in the preparation of the medium and the washing procedure of the cells cause these deviations.

It is obvious from the table that the cells of the Δ*nik* mutant have a considerably lower nickel content (4,000 per cell) compared to the other strains (15,000 in wild type cells, 17,000 in *oehox* and 13,000 in Δ*hoxH*) as expected. All other metal contents are higher in the Δ*nik* indicating an increased uptake possibly triggered by the lack of nickel.

From inspection of the cells at the light microscope and measurements at the Coulter Counter it was obvious that the cells of the Δ*hoxH* mutant had a larger volume (7.7 µm^3^ compared to 3.1 µm^3^ in the wild type cells). On the basis of a volume ratio of 2.5 the data obtained from the ICP-MS was corrected in Δ*hoxH*.

If comparing the corrected metal content per cell of the Δ*hoxH* (Table 1) it is obvious that most transition metal contents are close to those of the other strains except cobalt, which is significantly higher and which might indicate a higher uptake activity caused by the lack of the hydrogenase. Taken together the ICP-MS measurements yielded convincing numbers of metal atoms per cell that were all well in range with previously published data.

### Photosystem stoichiometry and number of photosystems per cell

**Figure 3:**
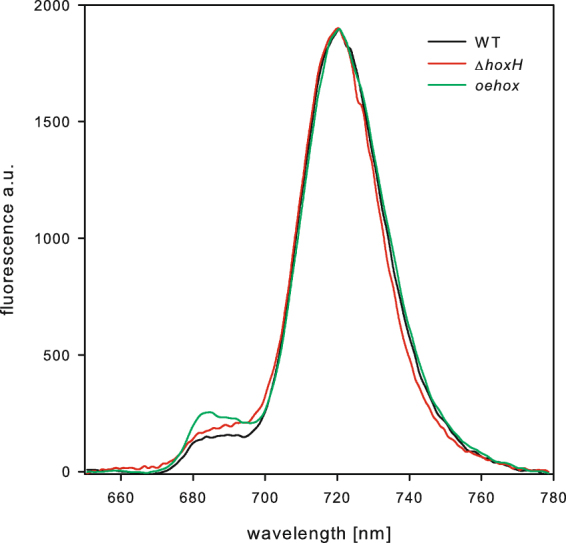
77 K spectra of wild type cells, *oehox*, and Δ*hoxH*. The peak at 720 nm originates from PSI and the two peaks at 685 and 695 nm correspond to CP47 and the reaction center of PSII, respectively. All the curves were normalized to the same fluorescence emission at 720 nm.

As we were interested to determine the ratio between hydrogenase molecules to PSI and PSII, the number photosystems per cell was determined. The chlorophyll content of the different strains (Table 2) and the 77 K chlorophyll fluorescence spectra (Fig. 3) were used to estimate the total number of photosystems per cell. According to established protocols a ratio of the photosystems can be approximated by deconvoluting the areas of their fluorescence maxima in the 77 K spectra^30^. Using this method we determined the ratio of PSI/PSII in wild type cells to 5.6 in the *oehox* to 3.6 and in the Δ*hoxH* as 3.8.

**Table 2:** Number of chlorophyll molecules and calculated PSII dimers and PSI monomers per cell.

Strain	Chl/cell	PSII dimers/cell	Mn/PSII/cell	PSI monomers/cell	Fe/PSI/cell
Wild type	3.65 ± 0.25 × 10^7^	2.36 ± 0.07 × 10^4^	1.89 ± 1.26 × 10^5^	2.68 ± 0.01 × 10^5^	3.21 ± 0.13 × 10^6^
*oehox*	4.53 ± 1.60 × 10^7^	3.36 ± 0.97 × 10^4^	2.68 ± 0.78 × 10^5^	2.50 ± 0.03 × 10^5^	3.00 ± 0.33 × 10^6^
Δ*hoxH*	7.16 ± 1.10 × 10^7^	5.15 ± 0.39 × 10^4^	4.12 ± 0.31 × 10^5^	5.21 ± 0.09 × 10^5^	6.25 ± 1.02 × 10^6^

On the basis of the recorded 77 K fluorescence spectra a PSI/PSII ratio of 5.6 was used for wild type cells, 3.6 for *oeHox* and a ratio of 3.8 for the Δ*hoxH* mutant. The number of chlorophyll molecules was reduced by 25% since this proportion is bound to small CAB-proteins^34^. 35 chlorophyll molecules per PSII^32^ and 96 per PSI^33^ were used on the basis of published X-ray structures to calculate the number of reaction centers.

The number of chlorophyll molecules per photosystem II reaction center is 35^31^ and 96 per photosystem I reaction center^32^. About 25% of the chlorophyll is bound to small CABs (chlorophyll a binding protein) in *Synechocystis*^33^. Based on this data it is possible to estimate the number of photosystems according to the formulas given in the materials and methods section. Wild type cells contain 23,600 PSII dimers (47,200 PSII reaction centers) and 268,000 PSI reaction centers (Table 2). This corresponds to 188,800 Mn atoms and 3.21 × 10^6^ Fe atoms. In all strains the metal atoms of the reaction centers amount to about half of the respective metal measured in the different strains (compare Tables 1 and 2).

### Number of NiFe-hydrogenase molecules per cell

**Figure 4:**
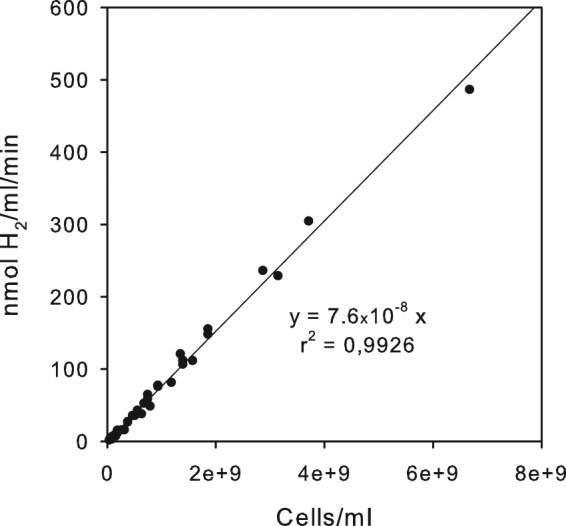
Hydrogenase acitivity as measured in the presence of 5 mM methylviologen and 10 mM dithionite of different amounts of cells. Cells from three independent wild type cultures were measured.

The hydrogenase activity assay used with 5 mM methylviologen and 10 mM dithionite allows activity measurements with whole cells. As shown in Fig. 4 the activity is linearly dependent on the number of cells added into the assay mixture over a wide range of cell numbers and could not be saturated even with about 6 × 10^9^ cells/ml. This correlation convincingly shows that the assay directly mirrors the amount of hydrogenase enzymes in the cells.

**Figure 5:**
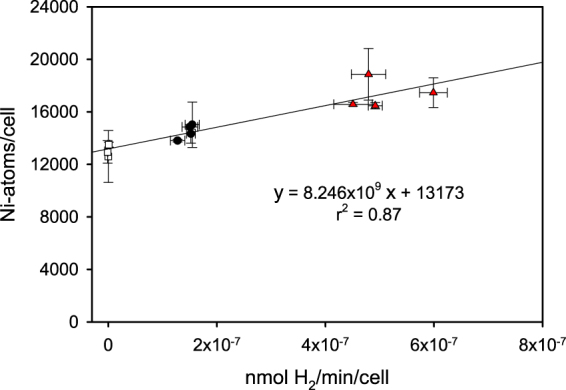
The number of Ni-atoms per cell plotted against the hydrogenase activity of Δ*hoxH* (open squares), wild type cells (filled circles) and the hydrogenase overexpression strain (*oehox*)(red triangles). Of each strain four independent cultures were measured at least in triplicates. Standard deviations are indicated.

When nickel content and hydrogenase activity per cell of wild type cells, *oehox*, and Δ*hoxH* were plotted against each other a linear relationship was obtained (Fig. 5). Since the correct insertion of the different metals into the different metalloenzymes requires a tight control of their content inside the cells^13,14^ we can thus assume that the different strains contain just the number of nickel atoms necessary for the number of active sites of their hydrogenase molecules besides a constant amount of other nickel dependent enzymes (e.g. urease) and nickel storage proteins (e.g. HypB1). It was possible to quantify the nickel pool that is not bound to the NiFe-hydrogenase by including Δ*hoxH* (corrected for its larger cell size) in our measurements. These Ni-atoms are not bound to hydrogenase molecules but to all other Ni-dependent enzymes. Since there is a single Ni-atom bound in the active site of the NiFe-hydrogenase we used this correlation to count the number of NiFe-hydrogenases in the cells according to their nickel content (corrected by the number of nickel atoms that are also present in Δ*hoxH* and therefore not bound to the NiFe-hydrogenase).

Ni-content and hydrogenase measurements correlated in a linear manner with a good statistical support with a correlation coefficient of 0.87. Using the derived equation we could therefore calculate that there are 1,100 ± 94 hydrogenase molecules per wild type cell and 3,790 ± 485 molecules in the cells of the overexpression strain.

To determine the variability of the number of hydrogenase molecules per cell and its ratio to the photosynthetic reaction centers we measured wild type cells from different culturing conditions and cell densities. Thereby, we obtained values of 400 to 2000 hydrogenase molecules per cell and ratios of one hydrogenase per 50 to 240 PSI centers and 40 to 55 PSII centers.

### Turnover frequency of the NiFe-hydrogenase

Based on the knowledge of the precise number of hydrogenase molecules per cell it is possible to determine the H_2_ turnover frequency per active site. Under the assay conditions with methylviologen as artificial electron donor and dithionite as reducing agent this turnover frequency is 1,340 ± 115 H_2_/s. This was calculated on the basis of the evolved H_2_ (Fig. 4) and the number of hydrogenase molecules in the wild type cells. Photohydrogen production is measured under physiological conditions without artificial electron donors but instead by illuminating anaerobic, dark-adapted cells. We determined turnover frequencies of NiFe-hydrogenases of different cyanobacterial strains when producing photohydrogen under *in–vivo* conditions using previously published data^20^. We first quantified the number of NiFe-hydrogenases on the basis of the available methylviologen and dithionite dependent measurements by means of the equation shown in Fig. 5 and then calculated the turnover frequencies based on the measured rate of photohydrogen production.

These calculations represent turnover frequencies of the NiFe-hydrogenase under physiological *in-vivo* conditions. Numbers are ranging from 18 H_2_/s for wild type cells to about 120 H_2_/s in case of the Δ*ctaI*Δ*cyd* mutant in normal BG-11 medium with nitrate (Table 3). The Δ*ctaI*Δ*cyd* mutant lacks the two respiratory terminal oxidases, the cytochrome c oxidase (*ctaI*) and the cytochrome bd oxidase (*cyd*) localized in the thylakoid membrane. These oxidases are able to withdraw part of the electrons from the light reaction even under anaerobic conditions due to the oxygen produced by PSII activity. Consequently the deletion mutant shows a higher photohydrogen production rate at the onset of light^20^.

**Table 3:** Turnover frequency for hydrogen production in the light in different strains.

Strain	Number of H_2_ases/cell	Photo H_2_ nmol H_2_/min/mg Chl	Turnover frequency H_2_/s
Wild type	430 ± 30	15.63 ± 2.50	18 ± 3
Δ*ctaI*Δ*cyd*	788 ± 42	187.50 ± 20.30	119 ± 13
Δ*cyd*Δ*ctaII*	830 ± 42	114.10 ± 17.20	69 ± 10
Δ*ctaI*Δ*cyd*Δ*ctaII*	670 ± 5	100.34 ± 18.80	75 ± 14
M55	62 ± 8	5.5 ± 0.40	45 ± 3

The cellular nickel content measured in this study allowed us to determine the turnover frequency of the NiFe-hydrogenase in wild type cells and some mutants of respiratory terminal oxidases on the basis of previously published data^20^.

In wild type cells the turnover frequency of the hydrogenase was dependent on the culturing conditions. The highest values of photohydrogen production of wild type cells were found at low cell densities (which corresponds to high photosynthetic activity) if the cells were shifted to nitrate free medium and directly measured. Under these conditions a maximum turnover frequency of the wild type cells of 62 H_2_/s was found.

For comparison the M55 mutant (45 H_2_/s), the mutant lacking all terminal respiratory oxidases (Δ*ctaI*Δ*cyd*Δ*ctaII*, 75 H_2_/s) and another double mutant (Δ*cyd*Δ*ctaII*, 69 H_2_/s) are also shown in Table 3. Since the M55 has no NDH-1 its cyclic electron transfer is very slow. It has a low hydrogenase activity and produces photohydrogen for several minutes^19,20^.

## Discussion

Cyanobacteria are known to harbor a number of different nickel dependent enzymes like the NiFe-hydrogenases, urease and SOD. The expression of these enzymes requires high affinity nickel uptake. Here we show on the basis of growth curves (Fig. 1) and activity measurements of hydrogenase and urease (Fig. 2) that the sole high affinity nickel transporter in *Synechocystis* belongs to a new type of energy coupling factor transporters and is encoded by *nikKLMQO* (sll0381 to sll0385). This result confirms predictions based on bioinformatics approaches^34^. In contrast to previous assumptions HupE is not able to complement the lack of this transporter^35^. Determination of nickel by ICP-MS of the hydrogenase deletion strain, wild type cells and a hydrogenase overexpression strain allowed the correlation of their nickel content and hydrogenase activity (Fig. 5). On the basis of this data we calculated about 1,100 hydrogenase molecules per wild type cell. Since we found 268,000 photosystem I and 47,200 photosystem II centers per cell on the basis of their chlorophyll content and 77 K spectra there is about 1 hydrogenase molecule per 240 PSI and 43 PSII reaction centers.

The high number of about 13,200 nickel atoms per cell not inserted into the active site of the hydrogenase (Fig. 5) suggests that the number of nickel dependent enzymes in *Synechocystis* might be higher than predicted on the basis of its genome sequence. Only the bidirectional hydrogenase, the urease and some other nickel binding proteins like the hydrogenase accessory genes HypB1, HypB2 and the urease accessory genes are known until now. Therefore, the actual metalloproteome of *Synechocystis* is probably underestimated as also shown for *Pyrococcus furiosus*^36^.

From this point on it is possible to also calculate the turnover frequencies of this NiFe-hydrogenase under different conditions and in different strains. In the presence of the artificial electron donor methylviologen we found the highest k_cat_ of about 1340 H_2_/s. This is in a similar range as the published value of the purified soluble hydrogenase (SH) of *Ralstonia eutropha*. The SH is closely related to the cyanobacterial bidirectional enzyme and has a k_cat_ of 372 H_2_/s when reducing ferricyanide with hydrogen^37^.

Highest rates of hydrogen turnover in *Synechcoystis* are achieved during photohydrogen evolution. The turnover frequency of wild type cells of up to 62 H_2_/s in the absence of nitrate still seems to be low since an oxidase mutant (Δ*ctaI*Δ*cyd*) even in the presence of nitrate shows about 120 H_2_/s (Table 3), which is up to now the strain with the highest rate achieved^19,20^. These results show that the hydrogen turnover is mostly dependent on the electron supply to the enzyme *in-vivo* and not limited by its characteristics.

The turnover frequencies of a variety of NiFe-hydrogenases has been measured. In this respect especially the SH of *R. eutropha* is interesting. For this enzyme a k_cat_ of 342 H_2_/s has been determined for the reduction of NAD^+^ with H_2_^38^. However, it has been shown by protein film voltammetry that the lower limit of the NiFe-hydrogenase of *Allochromatium vinosum* is 6,000 H_2_/s for the oxidation of H_2_^39^. These latter results suggest that the catalytic turnover of NiFe-hydrogenases can be significantly enhanced when it is possible to accelerate electron transfer rates to the enzyme.

It is very interesting to compare the turnover frequencies obtained in this study with those obtained by others using *in-vitro* constructs for light driven hydrogen production. Especially FeFe-hydrogenases are considered as highly efficient catalysts. Turnover frequencies of these enzymes of up to 21,000 ± 12,000 H_2_/s have been measured^40^. A FeFe-hydrogenase of *Clostridium acetobutylicum* tethered to PSI via a sulfhydryl linker showed a turnover rate of 105 ± 22 e^−^/PSI/s^22^ corresponding to about 53 H_2_/s. In another study it was shown that the membrane bound hydrogenase (MBH) of *R. eutropha* fused to the PsaE subunit of photosystem I yielded a rate of 75 ± 19 H_2_/s per complex when assembled on a gold electrode^23^. Thus, the rates of about 62 H_2_/s in wild type cells and 120 H_2_/s of the oxidase mutant measured here are comparable to and even higher than these systems working under optimal conditions.

The measured *in-vivo* turnover frequency of the bidirectional NiFe-hydrogenase of 120 H_2_/s in the oxidase mutant (Δ*ctaI*Δ*cyd*) is also above the *in-vivo* photosynthetic electron transfer rate of 47 e^−^/s estimated for PSI^22^ (equivalent to 24 H_2_/s) and even the rates measured *in-vitro* of 230 e^−^/s for isolated PSI (equivalent to 115 H_2_/s) saturated with ascorbate and ferredoxin^22^. Thus, the catalytic efficiency of the cyanobacterial NiFe-hydrogenase should not limit photohydrogen production when redirecting the cellular electron flow to this enzyme.

Our investigations expand previous electrochemical investigations^9^ and show that the bidirectional NiFe-hydrogenase of *Synechocystis* is a highly efficient catalyst for hydrogen evolution *in-vivo*. They show that metabolic engineering especially to direct photosynthetic electron transfer to this hydrogenase holds a great potential for high rates of biotechnological H_2_ production. The catalytic rates found here outcompete *in-vitro* systems poised at optimal redox potentials and working in the presence of plenty of electron donors. Since the cyanobacterial bidirectional NiFe-hydrogenase does not suffer from irreversible inhibition by oxygen and is partially active under low oxygen levels^9^ it holds an additional advantage compared to FeFe-hydrogenases. Moreover, *in-vivo* production systems would benefit from the self-repair inherent to living organisms.

## Material and Methods

### Growth conditions

*Synechocystis* sp. PCC 6803 wild type cells and the mutant strains were grown in BG-11^41^ supplemented with 5 mM TES pH 8. This medium contains a trace metal mix including Mn^2+^, Cu^2+^, Co^2+^, Zn^2+^ and MoO_4_^−^. The iron concentration in the medium is 22.9 µM. The mutant strains were segregated on BG-11 plates containing 50 µg/ml kanamycin. For physiological measurements cultures were grown in 200 ml BG-11 medium bubbled with air at a light intensity of 50 µE/m^2^/s. To deplete the cells of Co^2+^ and Ni^2+^ 1 mM nitrilotriacetic acid was added in some experiments as previously described^35^. At an OD_750_ between 3 and 4 the cells were harvested and subjected to all further measurements.

### Construction of mutants

To create a deletion mutant (Δ*nik*) of the putative nickel transporter genes PCR products containing the up- and downstream regions of the *nikKLMQO* (sll0381, sll0382, sll0383, sll0384 and sll0385) gene cluster and the kanamycin resistance cassette were prepared by PCR fusion as described previously^35^. The following primers were used P1nik CCGTGGGCAAAATCTACCCT and P2nik CTTTCTGGCTGGATGATGGGGCGATTAAAGGCGATCAGCAAACTGTGGG for the upstream region and P3nik TGTTGGAATTTAATCGCGGCCTCGAACCCACTTTTAAATGGCGAATCGC and P4nik ATCGTCAGGATCGTCTGCGG for the downstream region. The resulting PCR product was ligated into the pGEM-T vector (Promega, Madison, USA), checked by sequencing and transformed into *Synechocystis* sp, PCC 6803 wild type cells using established protocols^42^.

The construction of the deletion strain of the gene of the large hydrogenase subunit Δ*hoxH* was described previously^18^.

The strain overproducing the NiFe-hydrogenase was constructed by inserting the *psbAII-*promoter upstream of the *hox*-operon and the *hypFCDEAB*-operon form *Nostoc* sp. PCC 7120 under the control of the same promoter as described^43^. This strain retains the original structure of the *hox*-operon.

### Hydrogenase measurements

Hydrogenase was determined directly in whole cells as previously described using a Clark-type electrode pretreated for H_2_ measurements^20^. The electrode was connected to a lab-made control box maintaining a potential of −600 mV. An aliquot of the cells corresponding to 5 µg chlorophyll was given into the electrode chamber that was kept at a constant temperature of 28 °C. The chamber contained a total volume of 1 ml with the cells, 5 mM methylviologen and 10 mM sodium dithionite.

### Urease measurements

To measure the urease activity cells were pelleted by centrifugation and resuspended in a buffer with 100 mM Tris pH 8 and 150 mM NaCl. The cell suspension was vigorously vortexed with glass beads (diameter 0.17–0.18 mm) 3 × 3 min at 6 °C with intermittent cooling on ice. After centrifuging the suspension at 800 × g for 1 min at 4 °C the liquid phase was removed and centrifuged at 1,300 × g for 10 min to remove residual glass beads. An aliquot of this cell homogenate was used to determine the urease activity according to Kaltwasser and Schlegel^44^. The assay mixture contained in 1 ml 0.8 mM α-ketoglutarate, 0.03 M urea, 0.25 mM NADH and 9 U glutamate dehydrogenase and was buffered with 0.04 M Tris pH 8.0. The activity was measured by following the consumption of NADH at 366 nm with a spectrophotometer (Shimadzu UV-2501PC, Kyoto, Japan) at 20 °C.

### Determination of the cell number

The cell number and diameter was determined at least in triplicates with a Multisizer 3 (Beckman Coulter, Krefeld, Germany) with a capillary opening of 20 µm.

### Chlorophyll determination

A defined volume of cell suspension was pelleted by centrifugation for 5 min at 15,000 × g. The supernatant was removed. To extract the chlorophyll the cell pellet was vigorously vortexed with 1 ml methanol for 5 min. After an additional centrifugation at 15,000 × g for 10 min the supernatant was measured at a spectrophotometer at the wavelengths 665, 665.5, 666, 666.5 and 750 nm. The amount of chlorophyll was determined by taking the highest absorbance between 665 and 666.5 nm (Abs_high_) according to the formula [Chl a] = (Abs_high_ − Abs_750_)/0.0809 µg/ml^45^.

### 77 K fluorescence measurements

Chlorophyll fluorescence spectra at 77 K were recorded with a Hitachi F-4500 fluorescence spectrometer (Hitachi, Tokyo, Japan). The samples were diluted to 2 µg chlorophyll/ml frozen in liquid nitrogen and excited at 440 nm. The spectrum was recorded between 660 and 800 nm. By deconvolution of the spectrum it is possible to determine the ratio of PSI/PSII by using the areas of the different component bands^30^.

### Estimation of photosystems per cell

To estimate the number of photosystems per cell we used the total amount of chlorophyll measured in the cells, the cell number, the ratio of the two photosystems as determined from the 77 K spectra and the number of chlorophyll molecules per reaction center as given from the most recent X-ray structures^31,32^ according to the following formulas:

1 $$Number\,of\,PSI\,centers\,per\,cell=\frac{number\,of\,chlorophylls\,\times \,0.75}{cell\,number}\,\times \,\frac{\frac{PSI}{PSII}}{\frac{PSI}{PSII}\,\times \,96+35}$$

2 $$Number\,of\,PSII\,centers\,per\,cell=\frac{number\,of\,chlorophylls\,\times \,0.75}{cell\,number}\,\times \,\frac{1}{\frac{PSI}{PSII}\,\times \,96+35}$$

PSI/PSII is the ratio of the photosystems as determined from the 77 K spectra, where PSII was set to 1. The total amount of chlorophyll was reduced by 25% since this proportion of chlorophyll is bound to CABs (chlorophyll a binding proteins)^33^.

### Treatment of the cells for ICP-MS measurements

To analyze the different strains for their metal content their cell pellets of 150 ml culture were washed twice in 3 ml 0.2 M ultrapure EDTA pH 8. The pellet was resuspended in 3 ml distilled water and 700 µl of this suspension were pelleted again. This pellet was dried at 65 °C overnight and dissolved in 1 ml 69% aristar grade HNO_3_ and 500 µl ultrapure 30% H_2_O_2_. After three days in daylight the cell constituents were completely broken down by oxidation reactions. All steps and the harvesting were done in Greiner Bio one polypropylene tubes. An equal volume of BG-11 medium treated the same way was used as a control. For all cultures at least three replicates were prepared.

### ICP-MS

Samples were analyzed by inductively coupled-mass-spectrometry (ICP-MS) using a high resolution mass spectrometer (Thermo Element XR). All selected isotopes (^25^Mg, ^31^P, ^32^S, ^44^Ca, ^55^Mn, ^56^Fe, ^59^Co, ^60^Ni, ^63^Cu, ^66^Zn and ^115^In) were measured in medium ass resolution (4,000 RP) and carefully monitored for interferences. Prior to analysis samples were diluted 100fold and spiked with 2.5 ng/l In for internal standardization. Analytical results are blank-subtracted averages of three runs. Accuracy of the results was monitored by analyzing CRM’s NIST 1643e, NIST 1640 a and LGC6019 as unknowns. Average precision of results was estimated from replicate analyses of 12 samples and found to be better than <0.5% RSD for all elements except Ni with 2.5% RSD. Additional control measurements also including ^98^Mo were performed using quadrupole-based ICP-MS (Agilent 7500 cs) with lower sample dilution of 50fold, now giving a reproducibility of 0.8 and 0.3% RSD for Ni and Mo, respectively. A spiking experiment with 3 samples resulted in recoveries of >95% for all analytes.

### Data availability

All data generated or analysed during this study are included in this published article (and its Supplementary Information files).

## Electronic supplementary material

Dataset 1
