# Supplementary material for: In-vivo turnover frequency of the cyanobacterial NiFe-hydrogenase during photohydrogen production outperforms in-vitro systems
Source: Sci Rep. 2018 Apr 17;8:6083. doi: 10.1038/s41598-018-24430-y (PMC5904137; doi:10.1038/s41598-018-24430-y)
Supplement: Supplementary file 1 — Dataset 1 [file 41598_2018_24430_MOESM1_ESM.docx]

***In-vivo* turnover frequency of the cyanobacterial NiFe-hydrogenase during photohydrogen production outperforms *in-vitro* systems**

Kirstin Gutekunst^a^, Dörte Hoffmann^a^, Ulrike Westernströer^b^, Rüdiger Schulz^a^, Dieter Garbe-Schönberg^b^, Jens Appel^a*^

**Supplementary information**


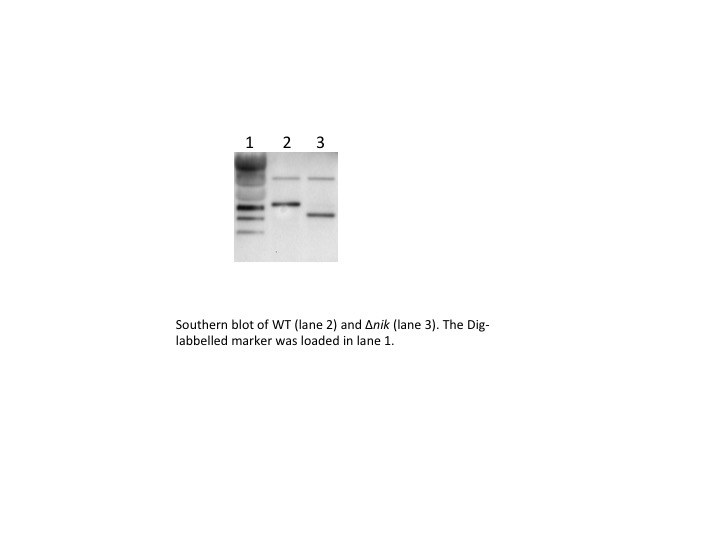


Fig. S1: Southern blot of WT (lane 2) and Δ*nik* (lane 3). The Dig-labelled marker was loaded in lane 1. The unspecific band is labelled with an arrow. In lane 2 and 3 100 ng of HindIII digested DNA have been loaded.


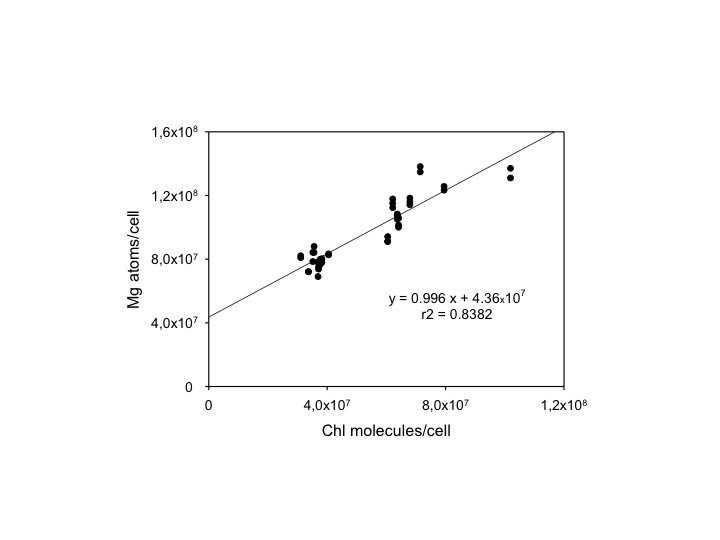


Fig. S2: Plot of the number of Mg atoms per cell against the number of chlorophyll molecules per cell of the strains used in this study. The y-axis intercept (4.36x10^7^) indicates the number of magnesium atom not bound to chlorophyll. On the basis of a cell volume of 3.1 µm3 this corresponds to a concentration of about 21 mM Mg^2+^.


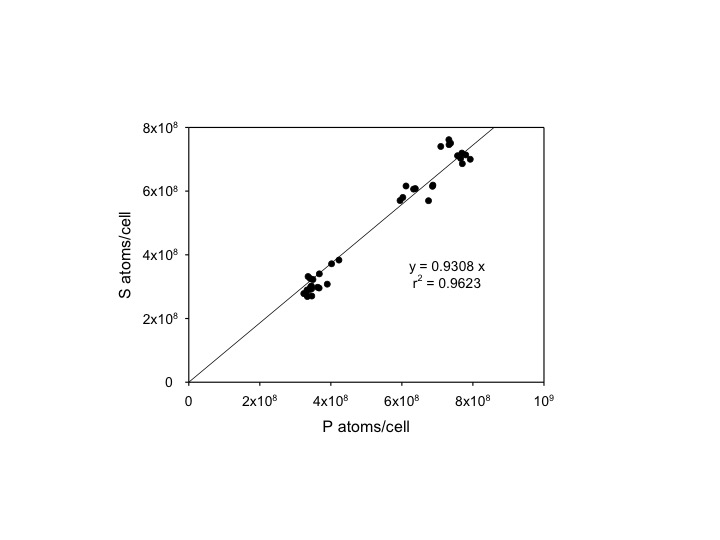


Fig. S3: The number of sulphur atoms plotted against the number of phosphorus atoms per cell of the different strains used in this study.
